# Large-Scale Wideband Light-Trapping Black Silicon Textured by Laser Inducing Assisted with Laser Cleaning in Ambient Air

**DOI:** 10.3390/nano12101772

**Published:** 2022-05-23

**Authors:** Zhidong Wen, Zhe Zhang, Kunpeng Zhang, Jiafa Li, Haiyan Shi, Man Li, Yu Hou, Mei Xue, Zichen Zhang

**Affiliations:** 1 Microelectronics Instruments and Equipment R & D Center, Institute of Microelectronics, Chinese Academy of Sciences, Beijing 100029, China; wenzhidong@ime.ac.cn (Z.W.); zhangzhe1@ime.ac.cn (Z.Z.); zhangkunpeng@ime.ac.cn (K.Z.); shihaiyan@ime.ac.cn (H.S.); liman@ime.ac.cn (M.L.); xuemei2020@ime.ac.cn (M.X.); 2 School of Integrated Circuits, University of Chinese Academy of Sciences, No. 19 (A) Yuquan Road, Beijing 100049, China; 3 Focal Plane Division, The 11th Research Institute of China Electronics Technology Corporation, Beijing 100846, China; ljf_ncrieo@126.com

**Keywords:** air, laser cleaning, micro-nano structures, light trapping, mid-infrared, large-scale

## Abstract

Black silicon, which is an attractive material due to its optical properties, is prepared mainly by laser inducing in an SF_6_ atmosphere. Considering the effect of SF_6_ gas on the environment and human health, here we propose an efficient, economical, and green approach to process large-scale black silicon. In the wavelength range of 0.3–2.5 µm, the role of air could replace SF_6_ gas to texture black silicon by laser inducing with appropriate processing parameters. Then, to extend the working window of its excellent light-trapping status, laser-plasma shockwave cleaning was introduced to eliminate the deposition and improve the structures and morphology. The results revealed that the micro-nano structures became higher, denser, and more uniform with increasing cleaning times and deteriorating cleaning velocity, which compensated for the role of S atoms from the ambient SF_6_. Moreover, absorptance above 85% in the wavelength range of 0.3–15 µm was realized using our method. The effect of scanning pitch between adjacent rows on large-scale black silicon was also discussed. Our method realized the ultrahigh absorptance of large-scale black silicon fabricated in air from visible to mid-infrared, which is of significance in the field of optoelectronic devices.

## 1. Introduction

With the boom in the field of solar-cell and optoelectronic devices, it is necessary to find ways to enhance light-trapping properties and energy conversion. The application of silicon is limited due to its bandwidth, and because its absorptance decreases abruptly when the light wavelength is over 1100 nm [1]. Black silicon, which is a silicon surface fabricated with micro-nano structures [2,3], is a promising candidate in research [4,5,6,7]. Since black silicon was first reported by Harvard University in 1998 [8], it has shown wide application potential due to its excellent absorptance and availability, such as in photocatalysis and photoelectrocatalysis [9,10,11], photodetection [12,13], solar cells [14,15,16,17], and sensing [18,19], etc.

However, given the processing performance, production efficiency, and cost, the fabrication of microstructures on a large-scale silicon surface is still a challenge for researchers and engineers. Several methods have been presented to process the microstructures on the silicon surface, including metal-assisted chemical etching (MACE) [20,21], wet chemical etching [22], reactive-ion etching (RIE) [23,24], and laser processing [25,26]. Laser technology demonstrates a prospective approach due to its flexibility, precision, and compatibility with CMOS processing. Previous research has mainly focused on laser processing in ambient SF_6_ gas [27] or other corrosive gases [28] due to hyperdoping of Si with S, which plays a role in enhancing infrared absorption. However, the industrial application of black silicon is limited considering its greenhouse effect, the cost of SF_6_ gas, and the complication of the laser equipment. Air is therefore a preferred choice, while laser processing requires improvement. Research focused on enhancing the performance of large-scale black silicon fabricated by laser inducing in ambient air is still lacking.

During the fabrication of large-scale black silicon induced by laser in air, a large quantity of particles deposit on the area around the line scanned by the laser. This affects the processing of the next line and the morphology of large-scale black silicon. In 2020, Tong Chen et al. [29] presented a method of laser ablation assisted with laser cleaning to remove the silicon oxide deposition and enhance the processing performance of the silicon surface in ambient air. Broad ultralow reflectivity was achieved experimentally in this research. However, the ablated square columnar structure remains a limitation of absorptance, and the ellipse spot used in the cleaning process makes the laser equipment more complex.

Laser cleaning is a novel surface-cleaning technology and exhibits attractive advantages [30], including environmental protection, noncontact, a wide range of applications, high accuracy, etc. With the development of ultrafast lasers, research related to laser-plasma shockwave cleaning has been conducted [31,32,33]. The mechanism and application of laser-plasma shockwave cleaning are still at the frontier of research.

To prepare ultrahigh-absorptivity black silicon surfaces at a large scale in a green and economical way, the method of laser inducing assisted by laser-plasma shockwave cleaning in ambient air is presented here. Laser-plasma shockwave cleaning was introduced to eliminate the oxide deposition induced by gravity and air. A number of experiments have been carried out, and the results, including the morphology and the optical properties, have been characterized to confirm the effectiveness of our method. Furthermore, to achieve a better performance for large-scale black silicon fabricated by laser inducing assisted with laser cleaning in ambient air, the effect of the processing parameters, including the laser-cleaning velocity, laser-cleaning times, and distances between adjacent rows were studied. This method provides an effective, green, and low-cost solution to the processing of large-scale black silicon, which extends the working window from visible light to mid-infrared. Our method is of significant importance to the industrial production of optoelectronic devices for broad wavebands, especially for solar cells, photodetection, and sensors.

## 2. Materials and Methods

**Figure 1:**
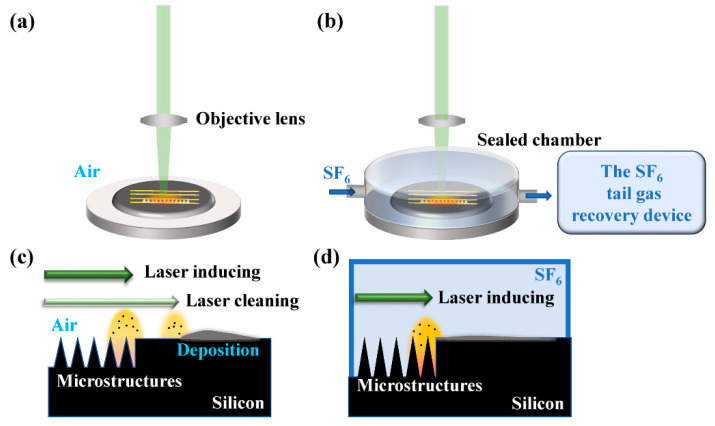
The schematic diagrams of the laser-processing equipment in (**a**) air and (**b**) SF_6_ gas. Illustrations of (**c**) the laser inducing assisted with laser cleaning in air and (**d**) the conventional inducing in SF_6_ gas.

In our experiments, N-type silicon wafers (100) with a thickness of 200 µm were available commercially to use as samples. The wafers were polished before laser processing. The 25 W laser source (NKT Photonics, Copenhagen, Denmark) used to fabricate the microstructures delivered 515 nm laser pulses with a duration of 400 fs and a repetition rate of 200 kHz. The position of the samples was controlled precisely by an x-y-z motion platform and its affiliated software. The schematic diagrams of the laser-processing equipment in ambient air and SF_6_ gas are illustrated in Figure 1a,b. The laser beam with a round laser spot of 40 µm was controlled through an objective lens (focal length of 75 mm) to irradiate the silicon surface in both the cleaning process and the inducing process. The samples processed in ambient air were attached to a loading platform, while those constructed in SF_6_ gas (pressure of 67 kPa) were in a sealed chamber. In addition, an SF_6_ tail-gas-recovery device was equipped given the influence of SF_6_ gas on the environment and human health. To fabricate a large-scale black silicon surface with micro-nano structures, several scanning trajectories with fixed spacing were designed with the software, which are the yellow rows in Figure 1a,b.

The schematics of our method in air and the conventional inducing process in SF_6_ gas are illustrated in Figure 1c,d. In our method, compared with conventional laser-inducing technology, the laser-plasma shockwave cleaning technology and laser induction were conducted rotationally. The scanning velocity was 20 mm/s with a laser influence of 1.8 J/cm^2^ during the inducing process. The process is elaborated as follows: Firstly, the first line was scanned using laser-inducing technology; secondly, cleaning technology was conducted along the next row to remove the deposition from the previous inducing process; thirdly, the line cleaned after the second step was scanned by the laser-inducing technology to form the micro-nano structures; and finally, the other rows were processed by repeating the second and the third step to form large-scale microstructures on the silicon surface.

The morphology of the processed samples was characterized by scanning electron microscope (SEM) (HITACHI, Tokyo, Japan) and laser scanning confocal microscope (OLYMPUS, Tokyo, Japan). The 3D optical images that capture the shapes of samples were obtained from the laser scanning confocal microscope to confirm the large-area morphology of the silicon surface and the RMS value. The color bar corresponded to the height of the model built in 3D images. The RMS value, which is the root-mean-square height for the evaluation area, was calculated to characterize the roughness of the surface. The correct height of the microstructure was obtained from cross-sectional SEM images of the samples, processed with the breaking process. The absorptance property was analyzed using an ultraviolet spectrophotometer (for the wavelength range from 0.3–2.5 µm) (SHIMADZU, Kyoto, Japan) and a Bruker Tensor–Fourier transform infrared (FTIR) spectroscope equipped with integrating spheres (for the wavelength range from 2.5–15 µm).

## 3. Results and Discussion

### 3.1. Formation of Microstructures Induced in Air and SF_6_ Gas

SF_6_ gas is a long-life greenhouse gas that has 2500 times the effect of CO_2_ gas on the environment. Moreover, the gas is also an asphyxiant and is harmful to human health in high concentrations. Air is an ideal environment for industrial production of large-scale black silicon given the greenhouse effect, cost, and harm to human health of SF_6_ gas, and the increased complexity of the necessary laser processing equipment.

**Figure 2:**
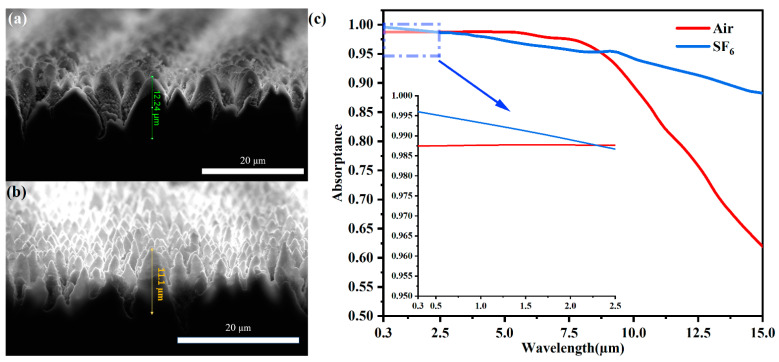
Cross-sectional SEM images and heights of conical microstructures of samples treated by laser inducing (**a**) in air and (**b**) in SF_6_ gas. (**c**) The absorptance spectra of (**a**,**b**). The rescaled inset highlights the absorptance curves in the wavelength range of 0.3–2.5 µm.

Figure 2a,b show that the conical microstructures are formed both in ambient air and SF_6_ gas, respectively. The heights of the conical structures on the black silicon surface fabricated in air are in the order of 12–14 µm, while the height of samples processed in SF_6_ gas is in the range of 10–12 µm. The width of structures processed in air is larger than those processed in SF_6_ gas. According to the reported simulation results, the reflectivity decreases with increasing height and increasing diameter of the conical structures when the diameter is lower than the periodicity [34]. Figure 2c illustrates the absorptance curves of samples processed in air and SF_6_ gas. In the wavelength range of 0.3–2.5 µm, the absorptance property of samples above 98% processed in the air was achieved experimentally using our optimized processing parameters, which is similar to the result of the sample treatment in ambient SF_6_ gas. The red line is higher than the blue one in the wavelength range of 2.5–9 µm. However, in the wavelength range from 8–15 µm, the absorptance curve of (a) is decreasing abruptly faster than the result of (b). When the light wavelength reaches 15 µm, the absorptance of the samples processed in air is about 62%, while it is 88% for the sample in SF_6_ gas.

There are three main infrared absorption mechanisms leading to the excellent optical properties of black silicon processed in SF_6_ gas, including free-carrier absorption, impurity photoionization, and indirect absorption [35]. (1) Free-carrier absorption: Free carriers absorb photons and transfer them from lower energy levels to higher energy levels; (2) Impurity photoionization: Superdoped sulfur impurity [36] leads to a wide continuous impurity band, which has a wide distribution of energy levels; (3) Indirect absorption: Micro-level conical structures exhibit a light-trapping property due to the multiple reflections of light between structures. It has been reported that indirect absorption plays a role mainly in absorption near the silicon-gap range. Impurity photoionization works in all the wavelength spectra, from short infrared wavebands to long wavebands. Free-carrier absorption dominates mainly in the long waveband. As seen in Figure 2a,b, the height of the structures fabricated in the air is close to the result of samples processed in SF_6_ gas. There is no sulfur impurity and fewer carriers in the samples constructed in ambient air. Consequently, in Figure 2c, the absorptance curve of (a) agrees well with the mechanisms reported in the research. In the wavelength region of 0.3–9 µm, the absorptance of (a) is close to the result of (b) because the high microstructures play a main role. With increasing wavelength, the effect of the sulfur atom and free-carrier absorption on the absorptance property grows, and the absorptance curve of (a) drops faster than the other curve.

The presence of SF_6_ gas hampers the production and wide application of black silicon material in the field of optoelectronics. Black silicon fabricated with appropriate processing parameters by laser inducing in the air could replace the method of processing in SF_6_ gas for optoelectronic devices, especially for the wavelength regions of visible and near-infrared, such as in light photodetection and solar cells.

### 3.2. Enhanced Performance in the Air Using the Method of Laser-Plasma Shockwave Cleaning

**Figure 3:**
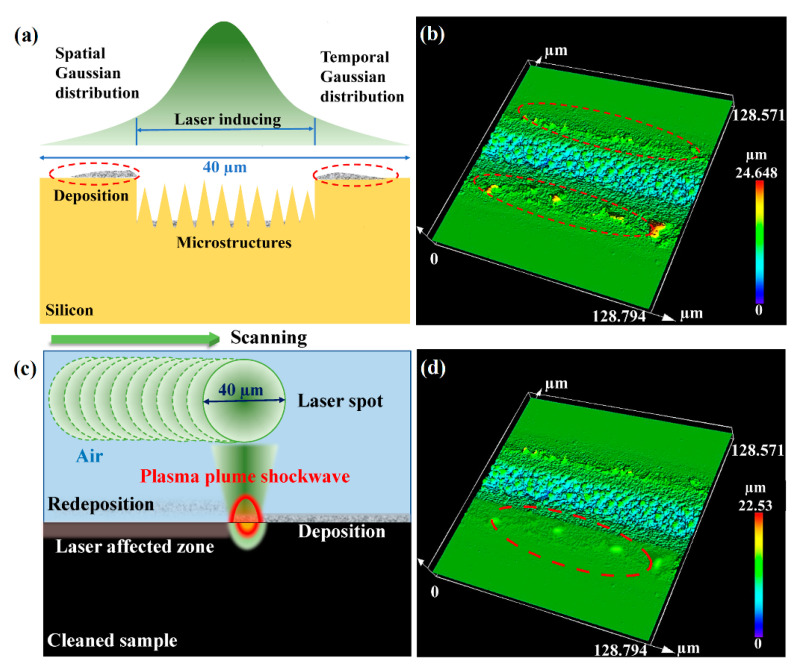
(**a**) The lateral schematic of the deposition produced in the laser-inducing process. (**b**) Top-view 3D image of a single line processed with the conventional laser-inducing technology. The deposition produced in the inducing process is highlighted in the red dotted circle. (**c**) The schematic diagram of the laser-plasma shockwave cleaning. (**d**) The result of (**b**) processed after the laser cleaning technology.

To further enhance the performance of black silicon processed in air and replace SF_6_ gas in the waveband of mid-infrared, the conical microstructures and morphology should be improved to enhance the light-trapping properties. During the conventional laser-inducing process in air, the particles splashed from the silicon surface deposit on the sides of the processing line due to a loss of kinetic energy [37]. Because of the existence of air, there are a large quantity of silicon oxides in the deposition. Figure 3a,b demonstrate the lateral schematic of the deposition produced from the laser inducing, and the top-view 3D image of a single line processed with conventional laser-inducing technology. The deposition affects the inducing result of the next processing line irradiated by the laser. Consequently, the optical properties of the large-scale black silicon are influenced, as well as the microstructures and morphology fabricated on the surface.

To eliminate the silicon oxide deposition produced by laser processing in air effectively and economically, laser-plasma shockwave cleaning was introduced to remove the nano-scale deposition. Laser-plasma shockwave cleaning is a novel cleaning method with the addition of ultrafast laser processing. Figure 3c indicates the schematic diagram of laser-plasma shockwave cleaning. The plasma plume and particles are produced under the irradiation of a femtosecond laser [38]. The phenomenon of gasification and ionization is performed in the plasma plume. The plasma expands with high pressure (>1 GPa) and a high temperature (>10^4^ K) abruptly. Finally, the laser-heated plasma explodes with the formation of the laser-supported detonation waves (LSDW) to remove the deposition [39]. The substrate should not be damaged during the cleaning process, though it will be modified by the shockwave and high temperature. The detailed mechanism of laser cleaning is still being assessed. Figure 3d shows the result of Figure 3b processed after laser-plasma shockwave cleaning to confirm the effectiveness of our method. In Figure 3b, the RMS value of the area remarked in red line without laser cleaning is 0.823 µm, while that value after laser cleaning at 20 mm/s for one time is 0.317 µm. With the removal of nanoparticles, a smoother surface is beneficial to induce higher microstructures by laser and to obtain a more uniform and higher morphology of large-scale black silicon.

In order to realize better performance of large-scale black silicon using laser-plasma shockwave cleaning in ambient air, the influence of parameters, including the cleaning velocity and the laser-cleaning times, is discussed. In 2020, Tong Chen et al. [29] reported that the ablation threshold of silicon oxide is less than that of monocrystalline silicon. Consequently, a proper laser fluence should be selected to remove silicon oxide deposition with no damage to the fabricated microstructures and silicon substrate. In our experiment, a laser-cleaning fluence of 0.3 J/cm^2^ was applied to remove the deposition produced from the previous processed trajectories.

**Figure 4:**
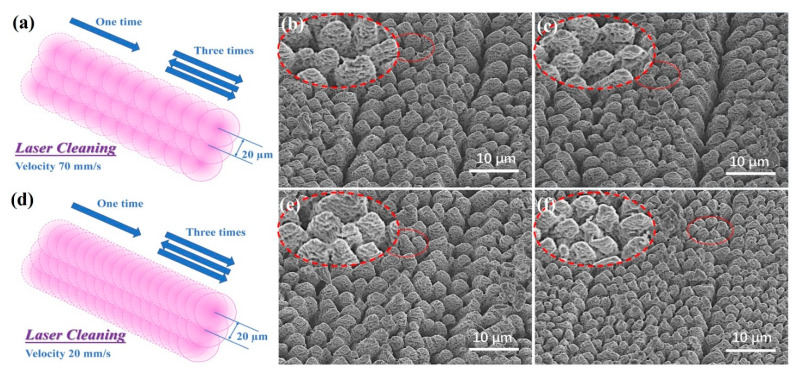
The schematics of overlapping laser spots with a scanning speed of (**a**) 70 mm/s and (**d**) 20 mm/s. Top-view SEM images of silicon samples processed with a cleaning velocity of 70 mm/s (**b**) one time and (**c**) three times, and the images of samples cleaned at 20 mm/s (**e**) one time and (**f**) three times. The red dashed circle enlarges the corresponding small circle area in each image.

**Figure 5:**
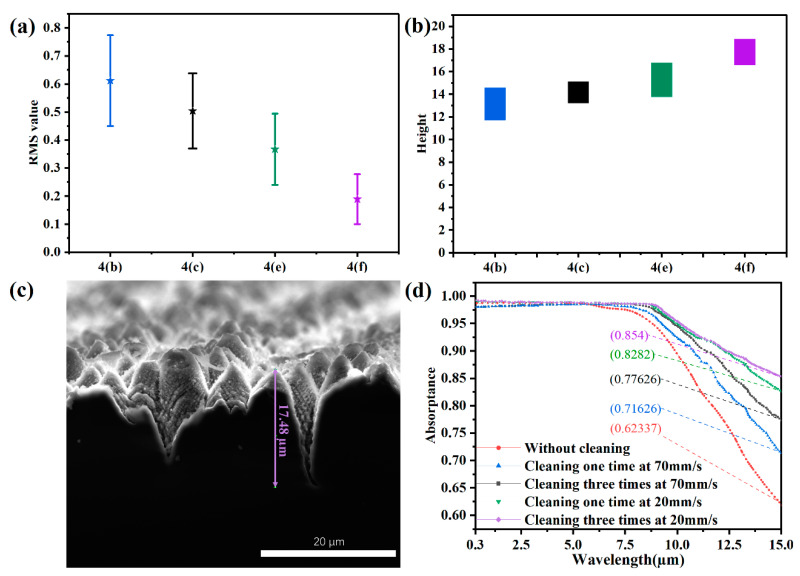
(**a**) The RMS values and (**b**) height ranges of the microstructures fabricated in Figure 4b,c,e,f. The error bar of the RMS value was calculated from 10 samples with each laser cleaning parameter. The height range was obtained from 20–30 data points on each sample surface. (**c**) The cross-sectional SEM image of samples processed with cleaning three times at 20 mm/s. (**d**) The absorptance curves of samples shown in Figure 4b,c,e,f and the sample induced without laser cleaning shown in Figure 2a.

Figure 4a,d show the schematics of overlapping laser spots with different scanning velocities in the cleaning process. In addition, cleaning three times means that the laser beam scans three times along the trajectory back and forth using laser-cleaning technology before the laser-inducing process. Figure 4b,c illustrate the top-view SEM images of silicon samples processed with laser cleaning one time and three times at 70 mm/s, respectively, while the results of samples cleaned one time and three times at the speed of 20 mm/s are shown in Figure 4e,f. The RMS values and the height ranges of samples processed with different laser-cleaning parameters are shown in Figure 5a and Figure 5b, respectively.

The RMS value showed a decreasing tendency with increasing cleaning times and decreasing cleaning velocity. The RMS value of the sample cleaned three times at 20 mm/s was 0.19 ± 0.09 µm, while the value without cleaning was about 0.823 µm. The results confirmed the effect of the laser-plasma shockwave cleaning times and velocity on removing nanoparticles. When the cleaning velocity was 70 mm/s, the height of microstructures processed with cleaning one time was in the order of 11.5–14.5 µm, and was 13.2–15 µm for the sample cleaned three times. With cleaning at 20 mm/s, the height of microstructures processed with cleaning one time was in the order of 13.5–16.8 µm, while it was 16.5–18 µm for the sample cleaned three times (cross-sectional SEM image shown in Figure 5c). With increasing cleaning times, the microstructures fabricated in the air became more uniform and higher. With the decreasing of cleaning velocity, the microstructures were higher and denser. It was also confirmed that the smoother silicon substrate modified by laser-plasma shockwave and high temperatures is helpful to fabricate higher and denser microstructures. Meanwhile, we found that the nanoscale textures grown on the conical microstructures were formed as demonstrated in the red dotted circle of Figure 4. The microstructure coated with nanotexture exhibited advantages in antireflection and excellent light-trapping properties [40]. The laser-plasma shockwave-cleaning technology eliminated the nanoscale deposition effectively, which is beneficial to inducing higher and denser microstructures on the next line. More uniform micro-nano structures fabricated on the large-scale black silicon surface were formed using our method.

It is well-known that the multiple reflections between the dense and uniform structures play a role in the ultrahigh light-trapping property of black silicon [41]. Figure 5d illustrates the absorptance curves of samples shown in Figure 4b,c,e,f, and the sample induced without cleaning. It is clear that the absorptance property of the samples was enhanced, especially in the region of mid-infrared, with increasing cleaning times and decreasing cleaning velocity. In the waveband of visible and near-infrared, the absorptance spectra of cleaning at 70 mm/s were close to the result without cleaning (0.98), and a slight increase (0.99) was observed for cleaning at a speed of 20 mm/s. In the waveband of mid-infrared, the results showed a slower decay with increasing cleaning times and decreasing cleaning velocity. At the wavelength of 15 µm, the corresponding absorptance property of each sample is shown in Figure 5d. Furthermore, the light-trapping property of samples processed with cleaning three times at 20 mm/s was enhanced to more than 85%, which is 1.37 times the result of samples processed without laser cleaning. To the best of our knowledge, the ultrahigh absorptivity shown in the samples processed with laser cleaning three times at 20 mm/s is the highest result treated in ambient air, compared with reported research on black silicon.

Increasing cleaning times provides an effective solution for the problem of redeposition of particles; the particles are vaporized due to the repeated high temperature and splashed due to the repeated high pressure of the shockwave. Different laser-scanning velocities result in different overlapping spot numbers at a particular spot, which combined with laser fluence leads to a different energy irradiation per unit area per time [42]. Additionally, at a slower cleaning velocity, the plasma plume accumulated at a higher temperature and higher pressure under femtosecond irradiation, which is helpful to remove the particles and modify the silicon substrate. The results revealed that the cleaning velocity has a bigger effect than the cleaning times on the absorptance property, as well as microstructures induced on the silicon surface. In addition, we confirmed that the role of multireflection between higher and denser micro-nano structures could compensate for the effect of sulfur atoms to some degree. However, given the production efficiency, appropriate cleaning times and cleaning velocity should be selected for the industrial production of large-scale black silicon in ambient air.

### 3.3. The Appropriate Scanning Pitch to Fabricate Large-Scale Black Silicon

**Figure 6:**
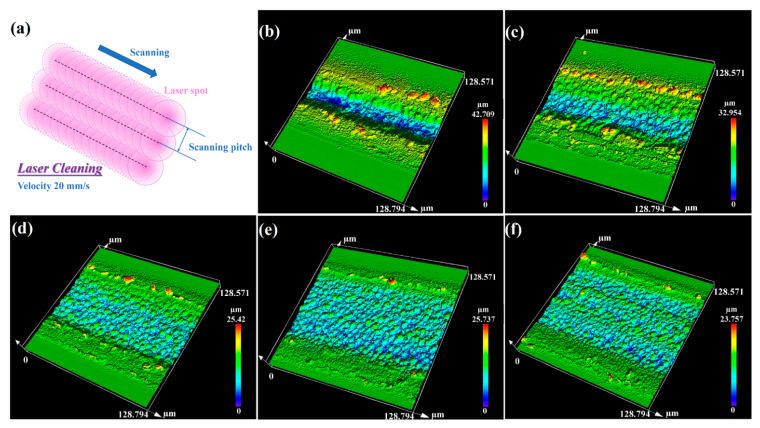
(**a**) The schematic of the scanning pitch during laser processing. The top-view 3D images of samples scanned for three rows at (**b**) 5 µm, (**c**) 10 µm, (**d**) 15 µm, (**e**) 20 µm and (**f**) 25 µm.

To meet the needs of commercial applications of black-silicon-based optoelectronic devices, there is a need to enhance the optical properties of large-scale black silicon fabricated in air. During processing, the fixed scanning pitch between adjacent rows is an important parameter for engineers to affect the morphology of large-scale black silicon; the schematic is depicted in Figure 6a. In our study, different scanning pitches (5, 10, 15, 20, and 25 µm) were discussed to achieve a better morphology, as well as absorptance properties. The 3D images of the samples scanned for three rows with different scanning pitches using our method in ambient air are presented in Figure 6b–f. As shown in Figure 6b,c, ablated pits formed obviously on the scanned area. Figure 6f shows that there are clear boundaries between adjacent processing lines. Consequently, it is obvious that more uniform microstructures were obtained with the distance of 15 and 20 µm, as shown in Figure 6d,e. The diameter of the laser spot in our study was 40 µm. In the fabrication of large-scale black silicon, too little a scanning pitch results in excessive overlapping energy above the ablation threshold on the irradiated area, while too large a scanning pitch leads to little overlapping laser energy to induce micro-nano structures on the sides of the processing lines and an undesirable morphology. Therefore, an appropriate scanning pitch, which was determined by the laser fluence, the diameter of the laser spot, the ablation threshold, and inducing threshold of the sample, is necessary for the laser processing of large-scale black silicon.

**Figure 7:**
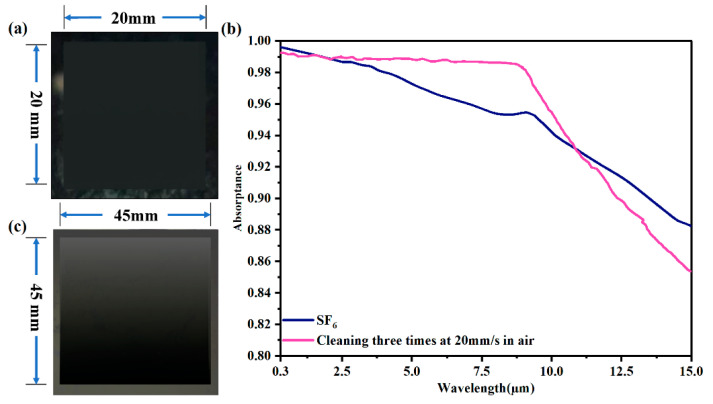
The top-view images of (**a**) 20 × 20 mm and (**c**) 45 × 45 mm large-scale black silicon samples fabricated using our method. (**b**) The absorptance curves of the 20 × 20 mm large-scale black silicon fabricated in air and the black silicon from SF_6_ gas shown in Figure 2b.

Furthermore, a 20 × 20 mm and a 45 × 45 mm sample of large-scale black silicon were prepared by laser inducing assisted with laser cleaning in ambient air, and the images are shown in Figure 7a,c. All processed lines except the first line were cleaned three times at a velocity of 20 mm/s before each laser inducing. The distance between adjacent rows was 20 µm. In Figure 7a, the area processed using our method is in black due to its ultrahigh absorptance and perfect light trapping. The area around the processed square is in grey in places due to the deposition and redeposition produced in the laser processing. The absorptance curves of the large-scale 20 × 20 mm black silicon fabricated in air and the black silicon processed in SF_6_ gas are illustrated in Figure 7b. In the wavelength range of 0.3–2.5 µm, the absorptance of the 20 × 20 mm sample was close to 99%, similar to the result in SF_6_ gas. In the waveband of 2.5–10 µm, the absorptance of the 20 × 20 mm sample was superior to the result from SF_6_ gas due to the main role of the microstructures. With increasing wavelength, the curve of the 20 × 20 mm sample decays faster because of the growing effect of sulfur atoms and free carriers. The results also proved that the role of multireflection between higher, denser, and more uniform structures could replace the effect of sulfur atoms to some degree. Therefore, a large-scale ultrahigh absorptance black silicon could be produced using our method, where the SF_6_ gas is replaced by air.

The method of laser inducing assisted by laser-plasma shockwave cleaning in ambient air was proved above to enhance the absorptance properties. Laser-plasma shockwave-cleaning technology is effective in removing the silicon oxide deposition to obtain higher, denser and more uniform micro-nano structures. Our method provides an excellent solution to the processing of large-scale black silicon for researchers and engineers, considering the performance of the treated samples, production efficiency, and cost. Attractive light-trapping properties produced in air were extended to the waveband of mid-infrared using our method, due to the improved micro-nano structures and morphology, which will be of use in the field of optoelectronic devices, such as infrared imagers and sensors. The ultrahigh-absorptance wavelength range of 2.5–10 µm coincides with that of the maximum black-body radiation from room temperature up to a few thousand Kelvin, giving it potential to realize ultracompact light sources with high efficiency [43].

However, the particles produced from the laser-plasma shockwave-cleaning process and the laser-inducing process may redeposit on the processed area. The effect of redeposition increases with the formation of larger areas of black silicon, with the processed area becoming grey, as shown in Figure 7c, which influences the morphology and the optical properties. Further research will need to be conducted to reduce the effect of the redeposition.

## 4. Conclusions

In summary, we have reported an economical, effective, and green method of laser inducing assisted by laser-plasma shockwave cleaning in ambient air to process large-scale ultrahigh absorptance black silicon. Laser-plasma shockwave cleaning was creatively introduced to remove the silicon oxide deposition produced in ambient air and improve the morphology of large-scale black silicon, as well as the absorptance. The following conclusions can be made:

(1) Given the greenhouse effect and cost of SF_6_ gas, ambient air is preferred for the industrial production of black silicon. In the wavelength range of 0.3–2.5 µm, the absorptance of black silicon induced with appropriate parameters in the air is ultrahigh enough to replace the role of SF_6_ gas. The microstructures play a main role in the wavelength range of 0.3–9 µm.
(2) To enhance the morphology and the absorptance of large-scale black silicon processed in air, the oxide deposition needs to be eliminated. The SEM images and RMS values show the effectiveness of laser-cleaning technology. The results indicated that the micro-nano structures become more uniform, higher, and denser with increasing cleaning times and reduced laser-cleaning velocity, which could compensate for the effect of sulfur atoms to some degree. In the wavelength range of 0.3–15 µm, absorptance above 85% was realized, which is the best result produced in air compared with previous research. Appropriate cleaning times and cleaning velocity should be selected in industrial production.
(3) The effect of scanning pitch on large-scale black silicon was investigated. Better morphologies were obtained with the scanning pitch of 20 µm and 15 µm in our study. An appropriate scanning pitch is determined by the laser fluence, the diameter of the laser spot, the ablation threshold, and the inducing threshold of the sample.

Considering the effectiveness, cost, and environmental impact, laser inducing assisted by laser cleaning in air is a preferred choice for researchers and engineers. Our method extends the ultrahigh absorptance of black silicon produced in air to the waveband of mid-infrared, which gives it wide application prospects in the development of optoelectronic devices based on black silicon for broad wavebands. In addition, it is beneficial to the evolution of laser equipment and multi-ultrafast laser processing. 

## Data Availability

The data presented in this study are available on request from the corresponding authors.
